# Plasma nuclear and mitochondrial DNA levels as predictors of outcome in severe sepsis patients in the emergency room

**DOI:** 10.1186/1479-5876-10-130

**Published:** 2012-06-21

**Authors:** Chia-Te Kung, Sheng-Yuan Hsiao, Tsung-Cheng Tsai, Chih-Min Su, Wen-Neng Chang, Chi-Ren Huang, Hung-Chen Wang, Wei-Che Lin, Hsueh-Wen Chang, Yu-Jun Lin, Ben-Chung Cheng, Ben Yu-Jih Su, Nai-Wen Tsai, Cheng-Hsien Lu

**Affiliations:** 1 Department of Emergency Medicine, Chang Gung University College of Medicine, Kaohsiung, Taiwan; 2 Department of Neurology, Chang Gung University College of Medicine, Kaohsiung, Taiwan; 3 Department of Neurosurgery, Chang Gung University College of Medicine, Kaohsiung, Taiwan; 4 Department of Radiology, Chang Gung University College of Medicine, Kaohsiung, Taiwan; 5 Medicine, Chang Gung Memorial Hospital-Kaohsiung Medical Center, Chang Gung University College of Medicine, Kaohsiung, Taiwan; 6 Department of Biological Science, National Sun Yat-Sen University, Kaohsiung, Taiwan; 7 Department of Neurology, Kaohsiung Chang Gung Memorial Hospital, Chang Gung University College of Medicine, No. 123, Ta Pei Road, Niao Sung Hsiang, Kaohsiung City 833, Taiwan

**Keywords:** Hospital mortality, Mitochondrial DNA, Nucleus DNA, Severe sepsis

## Abstract

**Background and aim:**

The sensitivity and specificity of biomarkers and scoring systems used for predicting fatality of severe sepsis patients remain unsatisfactory. This study aimed to determine the prognostic value of circulating plasma DNA levels in severe septic patients presenting at the Emergency Department (ED).

**Methods:**

Sixty-seven consecutive patients with severe sepsis and 33 controls were evaluated. Plasma DNA levels were estimated by real-time quantitative polymerase chain reaction assay using primers for the human β-hemoglobin and ND2 gene. The patients’ clinical and laboratory data on admission were analyzed.

**Results:**

The median plasma nuclear and mitochondria DNA levels for severe septic patients on admission were significantly higher than those of the controls. The mean plasma nuclear DNA level on admission correlated with lactate concentration (γ = 0.36, *p* = 0.003) and plasma mitochondrial DNA on admission (γ = 0.708, *p* < *0.001*). Significant prognostic factors for fatality included mechanical ventilation within the first 24 hours (*p* = 0.013), mean sequential organ failure assessment (SOFA) score on admission (*p* = 0.04), serum lactate (*p <* 0.001), and both plasma nuclear and mitochondrial DNA on admission (*p <* 0.001). Plasma mitochondrial DNA was an independent predictor of fatality by stepwise logistic regression such that an increase by one ng/mL in level would increase fatality rate by 0.7%.

**Conclusion:**

Plasma DNA has potential use for predicting outcome in septic patients arriving at the emergency room. Plasma mitochondrial DNA level on admission is a more powerful predictor than lactate concentration or SOFA scores on admission.

## Background

Severe sepsis and septic shock remain a great challenge in critical care because of their common occurrence, high costs of care, and significant mortality. They are the major causes of death in patients admitted to the emergency department (ED) and intensive care units (ICU), with mortality rates between 30% and 60% in different reports [1]. Numerous biomarkers for predicting morbidity and mortality in the critical care setting have been evaluated, but none have been proven entirely useful.

Plasma DNA can be defined as DNA fragments that are detectable in extracellular fluid and are of two types: “free” DNA present in plasma (including DNA packed into nucleosomes of apoptotic cells) or DNA associated with circulating lymphocytes (considered a minor component) [2]. Circulating plasma DNA has recently received increasing attention and has been studied in various acute and chronic disorders. Based on current evidence, DNA is released into the circulation from apoptotic and necrotic cells, although the exact mechanism is unclear [3,4]. An increase in plasma DNA concentration may be due to either increased liberation from cells or decreased clearance efficiency. Experimental animal studies have produced evidence suggesting that the liver and kidneys are the prime candidates for plasma DNA removal [5]. Rapid active liberation processes whereby DNA is released into plasma from either cell tissues or leucocytes are also possible. However, in critically ill conditions, organs responsible for elimination may be damaged as a consequence of ongoing systemic inflammation.

The early and high concentrations of plasma β-globulin DNA observed in various critical conditions, including trauma, stroke, myocardial infarction and septic shock, have been proposed as prognostic markers [6-11]. Apoptosis plays an important role in the patho-physiologic process of sepsis and circulating DNA has been detected in the plasma of septic patients [12]. Furthermore, fragmented DNA packed during apoptosis has been found in patients with severe sepsis [13]. Preliminary data from ICU and bacteremia patients suggest that admission plasma DNA concentrations may be higher in non-survivors than in survivors [14,15].

The aim of the present study was to investigate the prognostic value of circulating plasma DNA levels in patients with severe sepsis in the ED and intensive care setting.

## Patients and methods

### Study population and definition

This prospective study on the time course of plasma nuclear and mitochondrial DNA levels in severe sepsis and septic shock patients was conducted over a one-year period (January to December 2011). Sixty-seven adult non-traumatic patients at Chang Gung Memorial Hospital in Kaohsiung, a 2482-bed acute care teaching hospital that provides both primary and tertiary referral care, were enrolled. The hospital’s Institutional Review Committee on Human Research approved the study protocol and all of the patients provided written informed consent.

All patients aged >18 years consecutively admitted from the ED were screened daily for severe sepsis and septic shock according to specific criteria defined by the American College of Chest Physicians/Society of Critical Care Medicine. These criteria were suspected or confirmed infection, two or more manifestations of systemic inflammatory response syndrome, and at least one sepsis-induced acute organ dysfunction. Patients who met all three criteria were included [16].

For comparison, 33 age- and sex-matched healthy volunteers who received annual physical check-up and without clinical evidence of infection were recruited as controls.

### Clinical assessment and treatment

The medical records were prospectively recorded using pre-existing standardized evaluation forms that included demographic data, Acute Physiology and Chronic Health Evaluation (APACHE) II score, and the Sequential Organ Failure Assessment (SOFA) score, which was calculated during the first 24 hours of admission to assess the severity of organ dysfunction. Basic laboratory test, lactate concentration, B-type natriuretic peptide, and inflammatory markers (i.e., plasma C-reactive protein and procalcitonin) were taken on ED admission. Data on the source of infection and use of antibiotics were recorded.

The course of various organ dysfunctions and supportive treatments, including vasoactive and ventilator therapies and renal replacement therapies, were recorded. Physicians evaluated the association of existing organ dysfunction and severe sepsis daily. Severe organ dysfunction or organ failure was defined as SOFA score ≥3. Patients on chronic dialysis treatment on admission and those with severe chronic liver disease were excluded from the acute organ dysfunction assessment. It was also institutional practice to consult an infectious disease specialist for anti-microbial treatment according to treatment guidelines for different infectious etiologies during the first 24 hours, and the provision of hydrocortisone (300 mg/day) therapy for 4 days.

### Blood sampling and assessment of plasma nuclear and mitochondrial DNA

Blood samples were collected on presentation to the ED (Day 1). Follow-up blood samples studies were obtained on Days 4 and 7 after admission to the wards or ICU. Under minimal tourniquet pressure, blood was taken from the antecubital vein using a sterile 19-gauge needle syringe in a single attempt while the patients was in a sitting position for at least 10 min. Peripheral venous blood (3 ml) was collected into EDTA-containing tubes. Two milliliters of whole blood was drawn from a peripheral vein using a 0.9-mm needle, with the cuff pressure limited to 40 mm Hg. The other samples were kept in heparinized tubes (50 IU heparin/ml; Sarstedt, Numbrecht, Germany). Heparin was chosen as the anti-coagulant because of its superior results compared to ethylene-diaminetetra-acetic acid, citrate, and a special cocktail (132 mM ethylenediaminetetra-acetic acid, 0.7% hydroxychloroquine sulfate, and 20 U heparin/ml) in experimental conditions. Procedural details were as described previously [17,18].

To ensure cell-free plasma collection, EDTA-blood were initially centrifuged for 10 min at 3000 rpm, followed by separation into a 1.5 ml clear polypropylene tube with care not to disturb the buffy coat layer. The newly separated aliquot was centrifuged for another 10 min at 10,000 rpm. The upper portion of plasma was then removed by a Pasteur pipette and placed into a further clear tube and frozen at −20°C prior to extraction. DNA was extracted from 200-μl plasma samples using a QIAamp Blood Kit (Qiagen, Frederick, MD) via the “blood and body fluid protocol” based on the manufacturer’s instructions. The exact amount used was documented for calculation of DNA concentration.

Plasma DNA was measured with the β-globin and MT-ND2 genes by real-time quantitative polymerase chain reaction (RT-PCR) assay (Roche Lightcycler; Roche, Lewes, UK) based on continuous measurements of SYBR Green fluorescent dye that could bind to double-stranded DNA generated during PCR [17,18]. The β-globin gene was present in all nucleated cells of the body, while the MT-ND2 gene was specific to mitochondrial DNA. The β-globin PCR system consisted of amplification primers β- globin-354F (5′-GTG CAC CTG ACT CCT GAG GAG A-3′) and β-globin- 455R (5′-CCT TGA TAC CAA CCT GCC CAG-3′). The 101-base-pair amplicon was detected using primer sequences and verified in the Genbank database (accession number U01317) [6]. The MT-ND2 PCR system consisted of the amplification primers MT-ND2-156F (5′-CAC AGA AGC TGC CAT CAA GTA-3′) and MT-ND2-245R (5′-CCG GAG AGT ATA TTG TTG AAG AG-3′). The 90-base-pair amplicon was detected using primer sequences and verified in the Genbank database (accession number NC012920).

The generation of a plasma DNA standard curve was accomplished using human genomic DNA. The expression of quantitative results as kilogenome-equivalents/l was as described previously [17,18]. One genome-equivalent was defined as the amount of a particular target sequence contained in a single diploid human cell.

All tests were performed by a quality-controlled central laboratory at Chang-Gung Memorial Hospital. Concentrations of CRP were determined by enzyme immunoassay with a detection limit of <5 mg/dL (EMIT; Merck Diagnostica; Zurich, Switzerland), while PCT was measured using Enzyme-Linked Fluorescent Assay (VIDAS; bioMerieux; Ponte a Ema, Italy). Serum lactate levels were measured using a serum-based assay catalyzed by lactate oxidase (Vitros, Ortho Clinical Diagnostics, Rochester, NY).

### Statistical analysis

Data were expressed as mean ± SD or median (inter-quartile range). Univariate analyses were compared using Student’s t-test while categorical variables were compared using χ2 test or Fisher's exact test, as appropriate. Multiple comparisons among pairs of means were performed by multiple significant tests with Bonferroni method. Repeated measures of ANOVA were used to compare plasma cell-free DNA at three different time points after severe sepsis. Analysis of covariance (ANCOVA) was used to compare the groups after controlling for potential confounding variables.

Correlation analysis was used to explore the relationship among 24-h APACHE II score, SOFA score, Lactate, C-reactive protein, procalcitonin, creatinine concentration, and levels of plasma nuclear and mitochondrial DNA of severe sepsis patients on admission. Stepwise logistic regression was used to evaluate the relationship between significant variables and therapeutic outcomes, with adjustments for other potential confounding factors. Variables with zero cell count in a 2-by-2 table were eliminated from logistic analysis and only those strongly associated with fatality (*p* < 0.05) were included in the final model. Receivers operating characteristic (ROC) curves were used to estimate an optimal cut-off value for the use of both DNA nuclear and mitochondria DNA measurements for predicting death as well as the sensitivity and specificity of the test at this level. All statistical analyses were conducted using the SAS software package, ver. 9.1 (2002, SAS Statistical Institute, Cary, NC).

## Results

### Baseline characteristics of the study patients

The baseline characteristics of adult severe sepsis and septic shock cases (n = 67) and healthy controls (n = 33) showed no significant differences in underlying disease (i.e., hypertension, diabetes mellitus, and cardiac disease) (Table 1). The controls had median plasma nuclear DNA level of 25 ng/ml (range, 19–41 ng/ml) and median plasma mitochondria DNA level of 16 ng/ml (range, 7–23 ng/ml). The median plasma nuclear and mitochondria DNA levels for severe sepsis patients on ED admission were significantly higher at 436 ng/ml (range, 216–1140 ng/ml) and 149 ng/ml (range, 79–304 ng/ml), respectively (both *p <* 0.001).

**Table 1:** Baseline characteristics of severe sepsis patients and control subjects

	***Control n = 33***	***Study Patients n = 67***	***p value***
*Age (y) (mean±SD)*	*66.3± 8.0*	*64.8± 13.8*	*0.57*
*Male (%)*	*66.7*	*65.7*	*1.00*
*Underlying diseases*			
*Diabetes mellitus (%)*	*54.5*	*35.8*	*0.09*
*Hypertension (%)*	*60.6*	*44.8*	*0.20*
*Cardiac disease (%)*	*12.1*	*11.9*	*1.00*
*Systolic blood pressure (mmHg) (mean±SD)*	*143±15.2*	*112.2±42.3*	*<0.001*
*Diastolic blood pressure (mmHg) (mean±SD)*	*76.5±10.1*	*70.5±24.2*	*0.09*
*Laboratory data*^*†*^			
*Mean (SD) WBC (×10*^*9*^*/L)*	*6.8 (2.5)*	*15.9 (10.7)*	*<0.001*
*Mean (SD) Platelet (×10*^*4*^*/L)*	*234.4 (66.4)*	*158.2 (94.4)*	*<0.001*
*Mean (SD) Hemoglobin(mg/dL)*	*13.6 (1.7)*	*11.9 (2.2)*	*<0.001*
*Mean (SD) CRP (mg/dL)*	*1.2 (0.9)*	*189.6 (124.8)*	*<0.001*
*Median (IQR) plasma nuclear DNA (ng/mL)*	*25 (19,41)*	*436 (216,1140)*	*<0.001*
*Median(IQR) plasma mitochondrial DNA ( ng/mL)*	*16 (7,23)*	*149 (79,304)*	*<0.001*

Note: SD, standard deviation; IQR, inter-quartile range; †, laboratory data at presentation in the patients group.

In terms of source of infection and causative pathogens in septic patients by positive blood culture, 11.9% (8/67) had Gram-positive bacteria and 40.3% (27/67) had Gram-negative bacteria (Table 2). The most common primary site of infection was the pulmonary system (40.3%). The baseline characteristics of survivors and non-survivors in severe sepsis patients revealed that 65.7% (44/67) of patients had septic shock within 24-h of admission and 35.8% (24/67) had ventilator treatment within 24-h of admission (Table 3). There were significant differences in patients with mechanical ventilation within the first 24-h (*p* = 0.013), maximum 24-h SOFA score on admission (*p* = 0.04), lactate concentration on admission (*p <* 0.001), and plasma nuclear and mitochondrial DNA within the first 24-h of admission (*p <* 0.001) between survivors and non-survivors.

**Table 2:** Source of infection and causative pathogens in septic patients

	***Survivors n = 56 (%)***	***Non-survivors n = 11 (%)***	***Total n = 67 (%)***
*Primary site of infection [n (%)]*			
*Respiratory tract infection*	*20 (30.3)*	*7 (10.6)*	*27 (40.2)*
*Urinary tract infection*	*12 (18.2)*	*1 (1.5)*	*13 (19.4)*
*Intra-abdominal infection*	*13 (23.2)*	*1 (1.5)*	*14 (20.9)*
*Soft tissue infection*	*8 (12.1)*	*2 (3.0)*	*10 (14.9)*
*Unknown origin*	*3 (4.5)*	*0 (0)*	*3 (4.5)*
*Causative pathogens [n (%)]*			
*Escherichia coli*	*15 (22.4)*	*1 (1.5)*	*16 (23.9)*
*Klebsiella pneumoniae*	*4 (6.0)*	*1 (1.5)*	*5 (7.5)*
*Other Gram negative bacilli*	*5 (7.5)*	*1 (1.5)*	*6 (9.0)*
*Streptococcal species*	*3 (4.5)*	*1 (1.5)*	*4 (6.0)*
*Staphylococcal species*	*4 (6.0)*	*0 (0)*	*4 (6.0)*
*Negative culture*	*25 (44.6)*	*7 (63.6)*	*32 (47.8)*

**Table 3:** Prognostic factors of septic patients at the emergency room

	***Non-survivors n = 11***	***Survivors n = 56***	***Crude OR (95% CI)***	***p value***	***Adjusted OR (95% CI)***	***p value***
*Age (y) (mean±SD)*	*69.6±9.7*	*63.5±14.1*		*0.09*		
*Male/Female*	*8/3*	*36/20*	*0.68(0.16-2.84)*	*0.74*	*-*	*-*
*Underlying diseases [n (%)]*						
*Diabetes mellitus*	*5 (8)*	*19 (28)*	*0.62 (0.17-2.28)*	*0.51*	*-*	*-*
*Hypertension*	*5 (8)*	*25 (37)*	*0.97 (0.26-3.55)*	*1.00*	*-*	*-*
*Liver diseases/alcoholism*	*3 (5)*	*8 (12)*	*0.44 (0.97-2.04)*	*0.37*	*-*	*-*
*Chronic obstructive pulmonary disease*	*2 (3)*	*6 (9)*	*0.54 (0.09-3.11)*	*0.61*	*-*	*-*
*Stroke*	*3 (5)*	*9 (13)*	*0.51 (0.11-2.30)*	*0.40*	*-*	*-*
*Coronary artery disease*	*0 (0)*	*8 (12)*	*0.81 (0.72-0.92)*	*0.34*	*-*	*-*
*Cancer*	*4 (6)*	*10 (15)*	*0.38 (0.09-1.55)*	*0.22*	*-*	*-*
*Connective tissue disease*	*0 (0)*	*1 (2)*	*0.83 (0.75-0.93)*	*1.00*	*-*	*-*
*Chronic renal disease*	*0 (0)*	*3 (5)*	*0.83 (0.74-0.93)*	*1.00*	*-*	*-*
*Glasgow coma score (mean±SD)*	*13.1±2.4*	*12.7±3.7*		*0.76*		
*Shock within 24 hours [n (%)]*	*7 (11)*	*37 (56)*	*1.18 (0.30-4.54)*	*1.00*	*-*	*-*
*Ventilator treatment within 24 hours [n (%)]*	*8 (12)*	*16 (24)*	*0.15 (0.04-0.64)*	*0.013*	*-*	*-*
*Disease severity index (mean±SD)*						
*Maximum 24-h APACHE II score*	*20.9±3.8*	*18.9±6.9*		*0.36*		
*Charlson Co-morbidity Index score*	*4.2±2.8*	*2.9±2.8*		*0.18*		
*Maximum 24-h SOFA score*	*7.5±1.8*	*5.6±2.9*		*0.04*		
*Laboratory data on admission*						
*Mean (SD) white blood cells (×10*^*9*^*/L)*	*16.9 (12.6)*	*15.7 (10.4)*		*0.72*		
*Mean (SD) Hemoglobin(mg/dL)*	*12.7 (2.7)*	*11.8 (2.1)*		*0.24*		
*Mean (SD) Platelet (×10*^*4*^*/L)*	*114.0 (55.6)*	*166.9 (98.3)*		*0.09*		
*Mean (SD) C-reactive protein (mg/dL)*	*161.8 (107.0)*	*195.7 (128.2)*		*0.42*		
*Mean (SD) Glucose (mg/dL)*	*251.4 (218.8)*	*167.5 (94.5)*		*0.24*		
*Mean (SD) Creatinine (mg/dL)*	*1.7 (0.6)*	*2.2 (1.6)*		*0.36*		
*Mean (SD) Lactate (mmol/L)*	*62.5 (48.2)*	*30.2 (13.8)*		*<0.001*		
*Mean (SD) Procalcitonin(mg/dL)*	*18.1 (16.4)*	*34.0 (52.9)*		*0.33*		
*Mean (SD) B-type natriuretic peptide (pg/ml)*	*776.3 (422.4)*	*679.5 (523.9)*		*0.79*		
*Median (IQR) plasma nuclear DNA (ng/mL)*	*2240 (1040–4760)*	*400 (210–886)*		*<0.001*	*0.094*	*1.0 (0.99-1.0)*
*Median(IQR) plasma mitochondrial DNA (ng/mL)*	*520 (218–846)*	*121 (69–227)*		*<0.001*	*0.015*	*0.99 (0.99-0.999)*

Abbreviations: SD, standard deviation; IQR, inter-quartile range; APACHE, Acute Physiology and Chronic Health Evaluation; SOFA, Sequential Organ Failure Assessment.

The median length of hospital stay was 14 days (range, 9–30 days). Furthermore, the mean first 24-h nuclear and mitochondria DNA concentrations were higher in patients who received mechanical ventilation in the ED compared to patients who did not (1603 vs. 715; *p* = 0.016 and 380 vs. 183; *p* = 0.047).

### Time course of plasma nuclear and mitochondrial DNA levels

**Figure 1:**
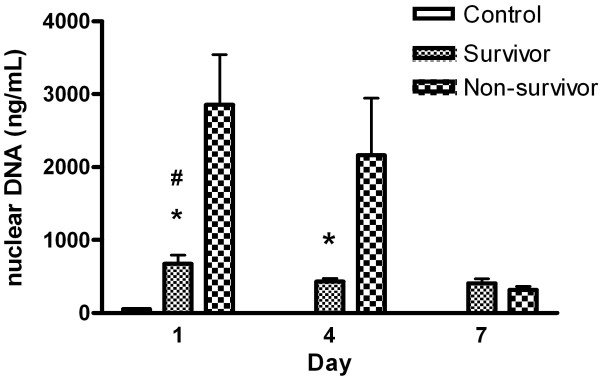
**Levels of circulating nuclear DNA in plasma on different days in severe sepsis patients and control subjects.**^#^*p* < 0.001, severe sepsis patients vs. controls; *p < 0.001, survivors vs. non-survivors.

**Figure 2:**
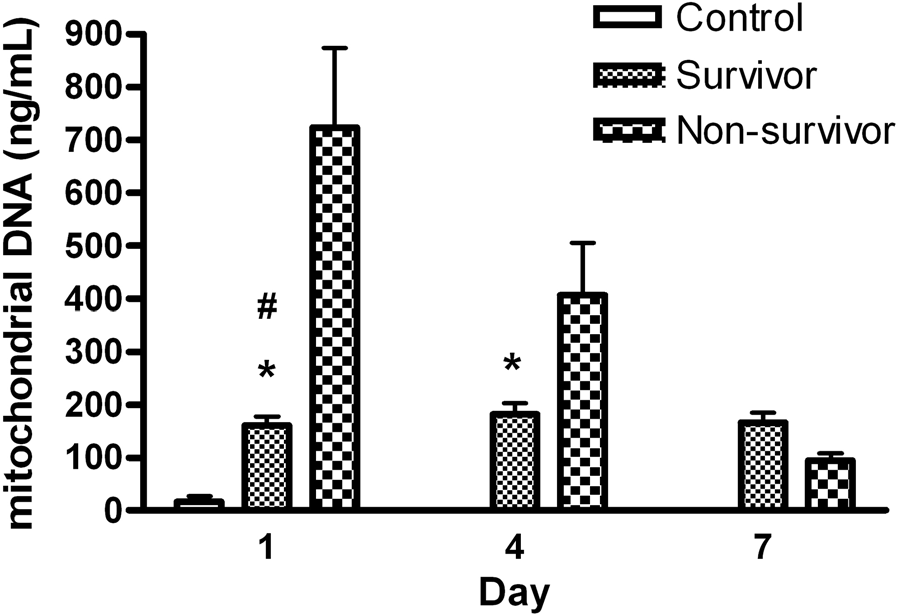
**Levels of circulating mitochondrial DNA in plasma on different days in severe sepsis patients and control subjects.**^#^*p* < 0.001, severe sepsis patients vs. controls; *p < 0.001, survivors vs. non-survivors.

Time course of circulating nuclear DNA and mitochondrial DNA in severe sepsis patients and control subjects were presented in Figure 1 and Figure 2, respectively. In patients with severe sepsis, the mean plasma nuclear DNA was significantly higher in non-survivors than in survivors on admission (2855 ± 2292 vs. 675 ± 904, *p* < 0.001) and 72-h later (2161 ± 2226 vs. 429 ± 255, *p* < 0.001). Mean mitochondrial DNA concentrations were also higher in non-survivors than in survivors on admission (723 ± 830 vs. 161 ± 128, *p* < 0.001) and 72-h later (406 ± 367 vs. 182 ± 129, *p =* 0.001). Although plasma nuclear and mitochondrial DNA levels decreased after anti-microbial therapy, there were no significant differences between survivors and non-survivors at three time periods (Days 1, 4, and 7) (*p =* 0.302 and *p =* 0.073).

### Correlation between plasma DNA and inflammatory biomarkers

By correlation analysis, the mean plasma nuclear DNA level on admission significantly correlated with lactate concentration (γ = 0.36, *p* = 0.003) and plasma mitochondrial DNA on admission (γ = 0.708, *p* < *0.001*). There was no significant correlation between plasma nuclear DNA and C-reactive protein (γ = 0.047, *p* = 0.705), N-terminal brain natriuretic peptide (γ = 0.005, *p* = 0.971), and procalcitonin (γ = −0.104, *p* = 0.971). There were also no significant correlations between plasma mitochondrial DNA and lactate concentration (γ = 0.164, *p* = 0.185), C-reactive protein (γ = −0.093, *p* = 0.457), N-terminal brain natriuretic peptide (γ = 0.014, *p* = 0.922), and procalcitonin (γ = −0.103, *p* = 0.432).

### Outcome and prognostic factors

**Figure 3:**
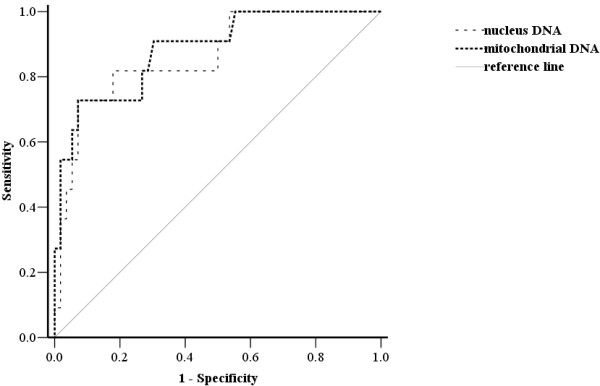
**The receiver operating characteristic (ROC) curve for both plasma mitochondrial and nuclear DNA levels on admission.** The area under the ROC curve is 0.883 and 0.864, respectively.

Eleven of the 67 (16%) patients died in the hospital, including 29 who received concomitant steroid therapy on admission for sepsis. The case fatality in steroid users and non-users was 17% (5/29) and 15.8% (6/38), respectively (*p* = 0.874). Potential prognostic factors of the 67 severe septic patients were listed in Table 3. Statistical analysis of the clinical manifestations and laboratory data between survivors and non-survivors revealed that the following were significant: mechanical ventilation within the first 24 h (*p* = 0.013), mean SOFA score on admission (*p* = 0.04), serum lactate (*p <* 0.001), and both the plasma nuclear (*p <* 0.001) and mitochondrial DNA on admission (*p <* 0.001). Of the variables used in the logistic regression (i.e., mechanical ventilation within the first 24 h, mean SOFA score on admission, serum lactate, and plasma nuclear and mitochondrial DNA on admission), only plasma mitochondrial DNA level was independently predictive of fatality, with a 0.7% increase in fatality rate per 1.0 ng/mL increase in level. The area under the ROC curve for both plasma mitochondrial and nuclear DNA level on admission was 0.883 (*p* < 0.001, 95% CI, 0.774-0.992) and 0.864 (*p* < 0.001, 95% CI, 0.773-0.984), respectively (Figure 3). The cut-off value for both plasma mitochondrial and nuclear DNA for predicting hospital fatality was 198 ng/ml (91% sensitivity and 72% specificity) and 1012 ng/ml (82% sensitivity and 82% specificity), respectively.

## Discussion

This study assessed serial circulating plasma nuclear and mitochondrial DNA levels in patients with severe sepsis and septic shock, and produced four major findings. First, both plasma nuclear and mitochondrial DNA concentrations on admission are significantly higher in severe septic patients than in healthy controls. Second, both plasma nuclear and mitochondrial DNA concentrations on admission are significantly higher in non-survivors than in survivors. Third, both plasma nuclear and mitochondrial DNA levels increase shortly after severe infection and gradually decrease after antimicrobial therapy. Furthermore, there were seven (7/11, 64%) fatal cases with imminent death within 7 days and their blood samples on Day 7 were not available. This may explain why the concentration rates of plasma DNA and plasma mitochondrial DNA were significant higher on Days 1 and 4 in the non-survivor group but similarly decreased in 7 days after treatment between the survivor and non-survivor groups. Lastly, significant prognostic factors for fatality include mechanical ventilation within the first 24 h, mean sequential organ failure assessment (SOFA) score on admission, serum lactate, and both plasma nuclear and mitochondrial DNA levels on admission, but only plasma mitochondrial DNA level is an independent predictor of fatality. Its cut-off value is 198 ng/ml (91% sensitivity and 72% specificity) and any increase of 1.0 ng/mL increases fatality rate by 0.7%. Plasma mitochondrial DNA level on admission is a more powerful predictor than lactate concentration or SOFA scores on admission, which are commonly used in clinical practice.

The present study shows that lactate concentrations on admission correlate independently with admission plasma DNA concentration. Both measures show significant differences between survivors and non-survivors, which may reflect similar mechanisms of septic shock-induced tissue hypoxia on apoptotic or necrotic cell death. Although the mechanisms underlying apoptosis in sepsis remain elusive, imbalance between oxygen delivery and consumption resulting in anaerobic glycolysis and lactate production are central features of severe septic infection. This may lead to oxygen deprivation, endothelial cell damage, and subsequent apoptotic cell death [19]. Previous studies indicate that circulating DNA originates from apoptotic and necrotic cells in cancer patients [4]. Huttunen et al. also showed that cell-free DNA expresses apoptotic fragmentation in bacteremia non-survivors [20], although the exact mechanism is not clear, reflecting the extent of cellular damage.

The present study reveals that circulating plasma nuclear and mitochondrial DNA concentrations are significantly higher in patients with mechanical ventilation on admission to the ED and in those who die within 6 days, and correlate highly with lactate concentrations. Thus, plasma nuclear and mitochondrial DNA concentrations are also good indicators of clinical severity in the ED. Rhodes et al. measured plasma nuclear DNA levels in 52 consecutive severely ill patients with or without severe sepsis or septic shocks who were in various categories of underlying conditions (i.e., traumatic, surgical, and non-surgical) and receiving heterogeneous treatments (i.e., surgical procedures, conservative treatment, or combined surgical and conservative management). However, only one blood sample was taken from each patient on admission. The study found that plasma nuclear DNA was higher in ICU patients than in healthy controls, and was higher in patients who developed sepsis or who subsequently died either in the ICU or in the ward [15].

In contrast, the current study enrolled non-traumatic, non-surgical severe septic patients at the ED patient. Both serial plasma nuclear and mitochondrial DNA levels were examined and demonstrated that both had potential use for predicting outcome in septic patients at the ED and plasma mitochondrial DNA level on admission is a more powerful predictor than lactate concentration or SOFA scores on admission. The discrepancy between the two studies may be attributed to different methodologies (e.g. patients on enrollment, heterogeneous treatments, and plasma DNA level), follow-up period (one blood sample vs. serial measurement), and statistical analysis methods.

Because the changing pattern of time course in plasma nuclear DNA is similar to that of mitochondrial DNA, both may originate from the same tissues by similar mechanisms. However, there is no correlation between plasma DNA concentrations and the three biochemical markers in this study used to assess prognosis (i.e., C-reactive protein, brain natriuretic peptide, and procalcitonin) in the ED. As such, the mechanisms necessary for the release of DNA are different from those implicated in the release of these biochemical markers. Several forms of sepsis-induced host cell death have been described during infection. The type of death the cell undergoes depends on the nature of the pathogen, pathogen load, and site of infection. Pyroptotic, apoptotic, autophagic, or oncotic cells display a distinct set of morphologic and biochemical characteristics [21]. Consequently, the source of free plasma DNA is complicated by the co-existence of several potential mechanisms of DNA release. Further prospective study is needed to clarify this relationship.

### Study limitation

Although the present study demonstrates that higher plasma mitochondrial DNA level on admission is a powerful biomarker for fatality in severe septic patients, this study has several limitations. First, plasma nuclear and mitochondrial DNA levels may be influenced by age and pre-existing diseases. Second, cellular damage indicated by plasma DNA may occur in different tissues and by several mechanisms of DNA release due to the heterogeneity of pathogens and critical illness of patients. Third, mechanisms of plasma DNA degradation and clearance also remain uncertain, particularly the impact of impaired renal and hepatic function on circulating DNA levels, which may be very complicated. Lastly, the choice of therapeutic strategy for sepsis (e.g., use of steroids, dosage and duration of steroids) may be different for each patient based on the preference of his/her doctor. This may cause potential bias in the statistical analysis. Large-scale prospective studies are warranted to evaluate the prognostic contribution of plasma DNA on clinical outcomes.

## Conclusion

Plasma DNA level has potential use in predicting the outcome of septic patients at the emergency room. Plasma mitochondrial DNA level on admission is a more powerful predictor than lactate concentration or SOFA score on admission, both of which are commonly used for outcome prediction in clinical practice. Cell-free DNA level in blood circulation is a novel biomarker for the prognosis of clinical diseases rather than diagnosis. The method has recently received increasing attention and its clinical application is expected. Based on the results, serial plasma DNA seems to meet the major requirement for outcome prediction in the treatment of severe sepsis. Plasma DNA assay can be considered a biomarker that should be added to the panel of conventional inflammatory biomarkers.

### Ethics approval

The study was approved by Chang Gung Memorial Hospital’s Institutional Review Committee on Human Research.

## Competing interests

The authors declare that they have no competing interests.

## Authors’ contributions

CTK participated in the design of the study and drafted the manuscript. SYH, TCT, CMS, WNC, CRH, and HCW participated in the sequence alignment and clinical evaluation of patients. WCL interpreted the imaging studies. YJL, BCC and BYJS performed the statistical analysis. NWT and CHL conceived the study, participated in its design and coordination, and helped draft the manuscript. All authors read and approved the final manuscript.
